# Public’s attitudes on participation in a biobank for research: an Italian survey

**DOI:** 10.1186/1472-6939-15-81

**Published:** 2014-11-26

**Authors:** Corinna Porteri, Patrizio Pasqualetti, Elena Togni, Michael Parker

**Affiliations:** Bioethics Unit, IRCCS Centro San Giovanni di Dio Fatebenefratelli, Via Pilastroni 4, 25125 Brescia, Italy; Service of Medical Statistics and Information Technology, AFaR Division, Fatebenefratelli Foundation for Health Research and Education, Lungotevere de’ Cenci, 5, 00186 Rome, Italy; The Ethox Centre, Nuffield Department of Population Health, University of Oxford, Old Road Campus, Oxford, OX3 7LF UK

**Keywords:** Public attitudes, Biobanks, Genetic research, Bioethics, Ethical policy

## Abstract

**Background:**

The creation of biobanks depends upon people’s willingness to donate their samples for research purposes and to agree to sample storage. Moreover, biobanks are a public good that requires active participation by all interested stakeholders at every stage of development. Therefore, knowing public’s attitudes towards participation in a biobank and biobank management is important and deserves investigation.

**Method:**

A survey was conducted among family members of patients attending the outpatient department of our institute for a geriatric or neurological visit, documenting their willingness to participate in a biobank and their views on the legal-ethical aspects of biobank management. Information regarding subjects’ attitudes on biomedical research in general and genetic research in particular was also collected. Participants’ data on biobanks were compared with data previously collected from the Italian ethics committees (ECs) to evaluate the extent to which lay people and ethics committees share views and concerns regarding biobanks.

**Results:**

One hundred forty-five subjects took part in the survey. The willingness to give biological samples for the constitution of a biobank set up for research purposes was declared by 86% of subjects and was modulated by subjects’ education. People in favour of providing biological samples for a biobank expressed a more positive view on biomedical research than did people who were not in favour; attitude towards genetic research in dementia was the strongest predictor of participation. Different from ECs that prefer specific consent (52%) and do not choose the option of broad consent (8%) for samples collection in a biobank, participants show a clear preference for broad consent (57%), followed by partially restricted consent (16%), specific consent (15%), and multi-layered consent (12%). Almost all of the subjects available to contribute to a biobank desire to receive both individual research results and research results of general value, while around fifty per cent of ECs require results communication.

**Conclusion:**

Family members showed willingness to participate in a biobank for research and expressed a view on the ethical aspects of a biobank management that differ on several issues from the Italian ECs’ opinion. Laypersons’ views should be taken into account in developing biobank regulations.

**Electronic supplementary material:**

The online version of this article (doi:10.1186/1472-6939-15-81) contains supplementary material, which is available to authorized users.

## Background

In recent years, biological banks have received much attention as a powerful tool to foster large-scale genetic research, which may increase our knowledge of human beings and hopefully will contribute to fighting diseases and improving the quality of human lives.

Laypersons’ involvement in the biobanking enterprise is crucial: the creation of biobanks depends upon subjects’ willingness to donate their samples for research purposes and to agree to sample storage; moreover, controversial projects of population biobanks have shown that not all biobank projects are warmly received by all groups in society and that biobanks are dependent on continual societal and political support to remain operational [1]. Finally and most importantly, biobanks are a patrimony for the present and future generations and should be regarded as a public good. This requires active participation of all interested stakeholders, including laypersons, at every stage of development. Laypeople’s experiences and perceptions need to count beyond the traditional expert opinion [2] to avoid incorrect definitions of problems and to allow a broad construction of our socio-technical future. Ways of linking biobanks with the views of citizens and patients should be researched [3, 4], and in fact a variety of approaches to increasing the role of public in the policy and governance of science and biotechnology has already been experienced [5], with the view of establishing a more pluralistic and inclusive style of policy making [6] that gives rise to a co-production of health and knowledge. For these reasons, knowing the public’s attitudes towards participation in a biobank and biobank management is important and deserves investigation.

In this paper, we present the results of a survey conducted among family members of patients attending the outpatient department of our institute for a geriatric or neurological visit, documenting their willingness to participate in a biobank and their views on the legal-ethical aspects of biobank management. A biobank for research on dementia and psychiatric disorders is set up in our institute. For this reason, the focus of our survey was on biobanks set up at a local level—such as in university departments, hospitals and scientific institutes—for purposes of research. Nevertheless, because the boundaries between different typologies of biobanks are somewhat flexible, our results may apply to some extent to biobanks in general. We also collected information about subjects’ attitudes regarding biomedical research in general and genetic research in particular to better understand our sample’s features and attitudes on different types of biomedical enterprise. Data on biobanks collected from participants, regarded as a sample of Italian citizens, were compared with data previously collected from the Italian ethics committees (ECs), to evaluate the extent to which laypersons and ethics committees share views and concerns on biobanks.

## Method

### Survey

A questionnaire-based survey was conducted among family members who were assisting patients attending a geriatric or neurological visit for cognitive impairment or dementia at the outpatient department of our institute. Family members of patients consecutively referring to the department were approached before their relatives’ visit and were asked for consent to participate in the survey after providing adequate information. None of the subjects approached had previously taken part in the constitution of a biobank. Only one member for family unit took part in the survey. Patients could not hear or interfere with the interview.

Three questionnaires were administered to the participants by a researcher not involved in the patients’ care or in biobank management, while subjects were provided with a copy of the questionnaires that they could follow during the interview to facilitate comprehension. Basic socio-demographic data were also collected. The survey aimed to register subjects’ attitudes; no offer of participation either in a biobank or in any other type of research project followed. The survey was carried out between May and October 2011.

The IRCCS Fatebenefratelli ethics committee approved the study.

### Questionnaires

The three questionnaires, named in the order of submission, were about participants’ attitudes towards: i) biomedical research, ii) genetic research, and iii) biobank participation and management.

The first questionnaire (9 questions) was translated into Italian from the Research Attitudes Questionnaire (RAQ) designed to measure how favourably or unfavourably one views biomedical research in general [7].

The second (8 questions) was a questionnaire that we developed to register participants’ views on genetic research: three questions aim to measure the attitude towards genetic research in general and specifically related to psychiatric disorders and dementia through a 5-points Likert scale; the others provide closed and exhaustive answers. The questionnaire was used for the first time in this study. Additional file 1.

The third questionnaire (17 questions) was adapted from one that we developed and used in a study about Italian ethics committees’ policies regarding the management of the ethical aspects of biobanks. The initial version of the questionnaire was tested for face validity through submission to five researchers and four ECs asked to comment on the clarity of the questions. A final version of the questionnaire was developed after the test and used in the study, demonstrating construct validity (e.g. ability to discriminate between ECs of scientific hospitals and of other clinical institutions). Forty-eight ethics committees (ECs) took part in that study. Except for the questions specifically related to ECs’ activities, that were omitted, the questions used in this survey were the same questions used in the previous study [8]. Only people expressing their willingness to take part in a hypothetical biobank completed the questionnaire on the ethical aspects of the biobank management, which was organised in the following areas: informed consent (IC) and information for the subject; protection of confidentiality; ownership of samples and data and intellectual propriety rights (IPRs); communication of research results; subjects’ remuneration and benefit sharing; access/transfer of biological materials and related data; and length of storage.

The time needed to complete the three questionnaires was from 15 to 50 minutes depending on the individual participant, with more than 50% of subjects completing the questionnaires in less than 20 minutes. The questionnaires were first administered to a group of ten persons to test their comprehensibility and to remove ambiguities. In particular, the second questionnaire was validated only as regard “face validity” and no psychometric procedures was applied before (e.g. criterion validity, test-rest reliability).

We observed that the first questionnaire (possible score for each question range from 1 to 5, with higher scores indicating a more favourable view) required multivariate analysis to obtain more meaningful scores. Therefore, the presence of distinct factors has been explored through factorial analysis (Principal Component Analysis).

## Results

### Participation in a biobank

One hundred forty-five subjects took part in the survey. On the basis of subsample analysis on 47 subjects, we can consider that they represented about 80% of all of the family members who were asked to participate. Their demographic characteristics are presented in Table 1. Age was symmetrically distributed around the average of 47.5 years, while females were clearly more represented among family members who were assisting patients attending a geriatric or neurological visit (75% vs. 25%). High school was the median education level.

**Table 1:** **Subjects’ socio-demographic features**

M/F ratio	36/109 (75%)
Age, years	47.5 (11.1)
Mean (SD)	
Education, years	13 (5–22)
Median (min-max)	

Factorial analysis of the first questionnaire allowed the identification of three factors, able to explain 53% of the total variance. Although this means that with three factors a large percentage of variance (47%) remains specific of single variables, Varimax rotation helped to clearly identify their meaning. The first could be labelled “utility of research” and was obtained by a linear combination of “discovery of cures for major diseases” (factor loading = 0.77), “personal utility” (factor loading = 0.63) and “protection of participants’ interests” (factor loading = 0.52). The second factor could be labelled “safety of research” and was obtained by combining “emphasis on research and harm” (factor loading = 0.81), “research is safe” (factor loading = 0.55) and “research does more good than harm” (factor loading = 0.53). The third factor could be named “boost of research” and was obtained by combining “positive view on research” (factor loading = 0.78), “need to devote more resources to research” (factor loading = 0.61) and (inversely) “research needs to be regulated by government” (−0.64). A total score, pulling together all the items of the questionnaire except “research needs to be regulated by government”, was also computed to have a unique index of “attitude to research” (the Cronbach’s alpha was 0.65, slightly below the conventional 0.70 threshold for adequate internal consistency).

The willingness to provide biological samples for the constitution of a biobank set up for research purposes was declared by 125 subjects (86%), while 20 (14%) were not available. Such large willingness was modulated neither by sex (p = 0.573) nor by age (p = 0.106) but by subjects’ education, with the percentage of those participating in a biobank rising from 69% among subjects with ≤8 years of school to 92% in those with 13 years of school and to 100% in those with a university degree (chi-square = 18.8, df = 2, p < 0.001).

**Figure 1:**
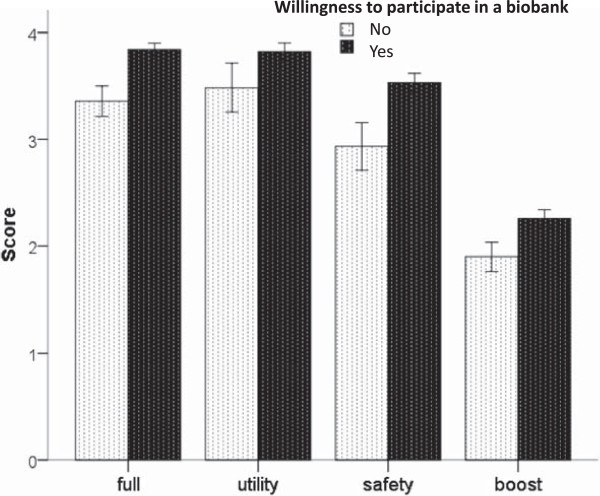
**Scores (means and 95% confidence intervals) on the full questionnaire and on the three subscales, according to willingness/unwillingness of providing biological samples for a biobank.** Higher scores were consistently observed in participants willing to biobank participation.

People in favour of providing biological samples for a biobank expressed a more positive view on biomedical research than did people who were not in favour, measured by the scores of both the full questionnaire and the three subscales (Figure 1). The differences between those in favour and those who were not in favour are quite similar in the three subscales (ANOVA, test for interaction, p = 0.151), although we observed the largest difference in terms of “safety of research”. To better address this point we applied a multiple logistic regression with “favour of giving samples for a biobank” as the dependent variable and the three subscales of the first questionnaire as covariates. We found that, even after adjusting for education effect, each subscale gave a significant contribution to the biobank participation (utility: OR = 3.9, 95% CI: 1.2-13.4; boost: OR = 5.6, 95% CI: 1.2-26.7; safety: OR = 8.2, 95% CI: 2.1-31.4). In other words, higher scores of each factor increased the probability of being available to participate in a biobank.

Subjects expressed a favourable attitude (possible scores for each question range from 1 to 5, with higher scores indicating more favourable views) toward genetic research in general (4.06, SD = 0.47) and genetic research related to psychiatric disorders (4.09, SD = 0.49) and dementia (4.16, SD = 0.54). Even if indexes of central tendency are consistently above 4, significant differences were found (Friedman nonparametric related samples ANOVA, p = 0.004), with scores of attitude toward genetic research in dementia being higher than the others (Bonferroni corrected p < 0.035). As expected, the scores of three attitudes toward genetic research are significantly correlated with each other (the lowest correlation was between attitude toward genetic research in general and genetic research in dementia, which was equal to 0.58, p < 0.001). However, according to logistic regression, scores of attitude toward genetic research in general does not “predict” the propensity to participate in a biobank (p = 0.109), while both scores of attitudes towards specific genetic research were more closely associated with biobank participation (p < 0.001). The multivariable analysis indicated that attitude toward genetic research in dementia is the strongest predictor of participation and its effect remains significant even taking into account education and scores of the first questionnaire: we estimated that for each 1-point increase the adjusted odd of being available to participate increased by 5.5 times (95% CI: 1.6-18.3).

Subjects also expressed a large willingness to take part in genetic research in general (77%), genetic research related to psychiatric disorders (75%), and dementia (77%), without any significant difference among them (Cochran test, p = 0.646). As expected, the relationship between such willingness and participation in a biobank was significant (p < 0.001 in all cases). In more detail, almost all of the subjects who were in favour of taking part in genetic research expressed an intention to participate in a biobank (>96%), while a percentage ranging from 44% to 53% of subjects who were not in favour of genetic research would participate in a biobank.

The major reasons for taking part in genetic research in general were to help in increasing knowledge and possible benefits for future generations (76%) as well as a possible benefit for a relative (42%). The major reasons for not being available for genetic research were, first, a fear of discovering possible genetic predisposition to certain diseases (49%)—this reason increases in research regarding psychiatric disorders (58%) and dementia (66%), and second, a worry that genetic materials may be used against personal principles (21%) or for commercial purposes (21%). Another important reason for not taking part specifically in research on psychiatric disorders and dementia was a worry that the genetic information may be used for discriminatory purposes (25% and 22%).

In addition, subjects were asked about their agreement on their relatives’ participation in genetic research on dementia: 105 subjects (72%) were in favour; those who were not in favour mainly showed a wish to protect the patient (42%) who was regarded as too old, not able to decide, or not able to benefit from the research.

### Opinion on the ethical aspects of biobanks

Regarding the ethical aspects of a biobank management, subjects answered as follows on the investigated areas. The full questionnaire was completed by 122/125 subjects. All percentages are expressed as percent of non-missing values.

**Table 2:** **Models of informed consent chosen by the public and by the Italian ECs**

	Public	ECs
	N =125	N = 48
Broad consent	71 (57%)	4 (8%)
Partially restricted consent	20 (16%)	18 (38%)
Multi-layered consent	15 (12%)	12 (25%)
Specific consent	19 (15%)	25 (52%)

Table 3 shows the type of information subjects (123/125 answered to this question) regard as important to receive when consent is collected. The comparisons between public and ECs are also reported.

**Table 3:** **Key information for informed consent: public and ECs opinion ordered from the more to the less important in the public’s view**

	Public	ECs	Fisher Exact test
	N = 123	N =48	***P-value***
Type of biological materials taken for a biobank creation	111 (90%)	44 (92%)	1.000
**Purpose for which biological materials will be used**	**110 (89%)**	**48 (100%)**	**0.021**
**Communication of research results**	**104 (85%)**	**21 (44%)**	**0.000**
**Prohibition of the commercialisation of samples and related data**	**98 (80%)**	**24 (50%)**	**0.000**
**Right to withdraw consent to the use of samples**	**98 (80%)**	**47 (98%)**	**0.002**
Right to withdraw consent to the use of data	97 (79%)	42 (89%)	0.275
Authorisation to be recontacted	86 (70%)	30 (63%)	0.367
**Rules to protect subjects’ privacy**	**83 (67%)**	**46 (96%)**	**0.000**
Independent ethics committee review	65 (53%)	18 (38%)	0.089
Transfer of samples and related data to other research institutes	65 (53%)	31 (65%)	0.175
**Authorisation to contact family members**	**62 (50%)**	**11 (23%)**	**0.001**
Access to samples and data from external researchers	59 (48%)	26 (54%)	0.499
Destiny of samples and data in case of advanced research conclusion	58 (47%)	25 (52%)	0.612
**Solidarity character of the participation**	**53 (43%)**	**36 (75%)**	**0.000**
Information on Intellectual Property Rights and patents	53 (43%)	20 (42%)	1.000
**Location of biological materials collection**	**48 (39%)**	**37 (77%)**	**0.000**
Destiny of samples and data after donor’s death	41 (33%)	17 (35%)	0.858
**Information on the property of samples**	**40 (33%)**	**25 (52%)**	**0.023**
**Duration of the storage**	**35 (28%)**	**32 (67%)**	**0.000**
**Benefit-sharing**	**0 (0%)**	**3 (6%)**	**0.021**

Significant (p < 0.05) differences between public and ECs were highlighted.

**Table 4:** **Communication of research results: public and ECs opinion**

	Research results of general value/yes		Individual results/yes		Fisher exact test
	Public	ECs	Public	ECs	p-value
	**N = 117/122 (96%)**	**N = 26/48 (54%)**	**N = 119/122 (98%)**	**N = 23/47(49%)**	
**Web site**	40 (34%)	4 (15%)			0.152
**Direct contact**	60 (51%)	12 (46%)			0.669
**Publication**	17 (15%)	14 (54%)			<0.001
**Any type of individual results**			45 (38%)	9 (39%)	1.000
**Only health relevant individual results**			74 (62%)	13 (57%)	0.645

P-value (Fisher Exact test) also reported.

**Table 5:** **Conditions expressed by participants for the access to samples and data collected in a biobank from others research organizations**

Conditions	%
Ethics committee’s opinion	34%
Use only for a specific research	7%
Destruction of the remaining biological materials	8%
Return of the remaining biological materials to the first biobank	18%
Impossibility for the organizations to identify the donor	37%
Constitution of a biobank	8%

1. Informed consent and information provided to the subjects
  Subjects were asked which type of IC for research on biological materials they would be willing to give (more than one option could be selected). The different types of consent [9] included the following options: (1) broad consent, which allows the use of biological specimens and related data for current research and future investigations of any type at any time; (2) partially restricted consent, which allows the use of biological specimens and related data for specific current research and future investigations directly or indirectly associated with current projects; (3) multi-layered consent, which requires several options to be explained to the research subject in a detailed form; and (4) specific informed consent, which allows the use of biological specimens and related data only for current research and forbids use for any future study that is not foreseen at the time of the original consent.
  Participants showed a clear preference for broad consent (57%), followed by partially restricted consent (16%), specific consent (15%), and multi-layered consent (12%) (Table 2). The comparisons between the public and ECs indicate clear differences for each type of consent, with public participants favouring broad consent (Fisher’s exact test, p < 0.001) and ECs favouring the other types of consent (p = 0.002 for partially restricted, p = 0.059 for multi-layered, p < 0.001 for specific). It should be noted that the answers were not mutually exclusive; thus, the percentages for each column can sum above 100% (although public participants chose only one answer) and, accordingly, statistical tests were applied for each type of consent. A large majority of participants (86%) believe informed consent should be given in a written form, while the others regard oral informed consent as also acceptable, whether documented or not. Subjects who do not choose broad informed consent want to be contacted again for the use of their samples in investigations not included in the original IC form (93%).
  In participants’ views (122/125), withdrawal of consent from the biobank should lead to sample destruction (58%), sample anonymisation (25%), or prohibition on using the sample in new research (7%).
2. Protection of confidentiality
  Regarding samples and data processing within the centre that collected them to protect subjects’ right to privacy, participants prefer the use of a code that makes it possible to later determine the donor’s identity (97%) to the anonymous use of samples (i.e., the irreversible removal of the link with the donor).
3. Communication of research results
  People who are ready to donate biological materials for biobank constitution also wish to be informed about the research results, both of general value (96%), through direct contact or publication, and of individual interest (98%), with a preference for results relevant to the donor’s health or family members’ health. Participants’ attitudes on the topic and those of ECs are compared in Table 4. Forty-three subjects (35%) want their research results to also be communicated to family members; 8 (19%) of them ask for communication to family members only if results are relevant for health.
4. Access/transfer of biological materials and related data
  Subjects were asked about access to samples and data by researchers who are not related to the centre that collected the biological materials. The large majority of participants regard access to samples by not-for-profit organisations (86%) as permissible, while only a minority (30%) agrees with access from for-profit organisations. In participants’ view, the access/transfer of biological materials and related data to both not-for-profit and for-profit organisations should be subjected to conditions, mainly regarding the impossibility of these bodies to identify the donor (37%) and the favourable opinion of the relevant ethics committee (34%) (Table 5).
5. Propriety of samples, IPRs and remuneration
  The majority of subjects believe the research/biobank sponsor should be the owner of the samples (69%) and should be entitled to intellectual propriety rights (IPRs) (67%), while donors should maintain sample propriety and IPRs respectively for 21% and 2% of the subjects. For 25 subjects (20%) IPRs should be shared between sponsor and donors, and for 7% of the subjects the income derived from the use of biological samples should be reinvested in research.
  Regarding donors’ remuneration to take part in a biobank, the large majority (87%) think donors should not be remunerated for taking part in a biobank; the others agree with remuneration, but exclusively as a flat refund and refund for documented expenses.
6. Duration of the storage
  The majority (51%) of subjects who answered the question do not care about the duration of sample storage and data preservation, while 36% express a preference for an open-ended period, and 13% for a limited period.

## Discussion

The large majority of subjects who were asked to participate in the survey gave consent. Moreover, people who decided not to participate provided reasons more related to logistics than to ideological issues. This large participation is an initial demonstration of laypersons’ wish to take an active part in the construction of knowledge. It also assures that our sample’s opinion is representative of the target population, i.e. family members of patients suffering from dementia or cognitive impairment.

The percentage of participants who expressed the willingness to provide biological materials for the constitution of a biobank is among the highest registered in attitude surveys [10]. The study was related to a hypothetical willingness of participating in a biobank: a request for real involvement in a biobank constitution could provide a different, but not necessarily lower, rate. Studies suggest factual willingness to participate in biobank research may be greater than hypothetical, in particular when donors are recruited in health care facilities and are possibly motivated by factors that are less influential in the hypothetical context, such as altruism, trust and sense of duty [10]. In our study, the aspect of hypothetical participation and the element of motivation due to subjects’ specific situation are combined.

The willingness to participate in the constitution of a biobank was modulated by subjects’ years of schooling, as other surveys found [11], confirming the common, although controversial, idea that higher education contributes to giving people more confidence in science.

Results show that subjects’ attitudes towards biomedical research can predict their attitudes towards participation in the constitution of a biobank, with people expressing a more positive feeling on biomedical research, and particularly on the safety of research, being more available to personally take part in a biobank. The result is also true regardless of the subject’s years of schooling, suggesting that not only education but also individual feeling, i.e., something not necessarily related to formal education or scientific knowledge, counts in expressing this choice.

The attitude towards genetics research on dementia was the strongest predictor of subjects’ participation in a biobank, even taking into account subjects’ education and feelings about biomedical research as expressed in the first questionnaire. These data support the intuitive perception that personal proximity to a disease and the suffering it causes the patients and their relatives makes people more aware and sensitive to the need for research and is the strongest motivation to accept direct involvement in research. The active promotion of research by patients and family members’ associations confirms this point [12].

People who were not available for genetic research manifested a fear of discovering a genetic predisposition to certain diseases, particularly to dementia. This indicates a potential conflict among the family members of people affected by a disorder: on the one hand, there is an increased motivation in contributing to the advancement of knowledge and the possibility of cure and, on the other hand, there is an increased fear of discovering a personal predisposition to the disease. We believe that the fact that psychiatric disorders and dementia are very often the object of social stigma [13] contributes to the worry that genetic information may be used for discriminatory purposes.

The percentage of people who were not available for genetic research but expressed willingness to take part in the constitution of a biobank may perhaps be explained by the idea that giving a sample to a biobank is less demanding than being actively involved in research. Anyway, a misunderstanding of the typical type of research carried out in a biobank cannot be excluded.

Our work was also designed to collect lay subjects’ opinion on the ethical and legal aspects of the management of biobanks and to compare their views with experts’ view, namely, with ethics committees’ opinions. The results showed that on several issues the Italian ECs and our sample of the public did not share the same view, even though the ECs are made up of members with different expertise, including patients’ representatives [14], and may be regarded, at least from an ideal perspective, as an expression of civic participation in the scientific enterprise.

The most relevant result concerns the model of IC and the pertinent information chosen by the ECs and the public. Informed consent is the most crucial and discussed element in the constitution of biobanks [15], where biological samples are collected at a certain point in time and then used for a long period of time, with research methods and opportunities rapidly evolving, which makes it difficult to give the subjects full information at the time of sample collection. The marked difference between the type of IC for research on biological materials that ECs regard as suitable and the type of IC that family members are willing to give may be partially due to a different awareness of the complexity of the ethical and legal issues related to IC among lay people and ECs. This interpretation seems to be confirmed by lay people’s tendency not to choose the more complex model of IC, namely, multi-layered consent. Nevertheless, public’s preferences cannot be simply ascribed to a lack of knowledge and awareness or to the desire not to be too much involved in demanding activities. On the contrary, the public seems to be more in line with researchers’ common idea that broad consent is essential to facilitate research opportunities and therefore scientific advancement. Other studies investigating different consent options found that most people are willing to provide one-time general consent for research [16], but different opinions were also registered [17]. The findings of the Eurobarometer survey EB 73.1 ‘Life Sciences and Biotechnology’, which took place in 2010 and contained questions on biobanks [18], showed that the option of broad consent appears not to be supported by the general public in Europe. People were invited to say which kind of permission they thought was needed when a scientist does research on data in a biobank among: not need to ask for permission (unrestricted consent), ask for permission only once (broad consent), ask for permission for every new piece of research (specific consent), don’t know. Data related to Italy show that 7% of the public thinks there is no need to ask for permission, 21% is in favour of giving permission only once, while 58% is in favour of giving permission for every new piece of research. Interestingly, attitudes in Europe towards broad consent are also shaped by the levels of information, with people who know more about biobanks being more willing to give broad forms of consent [11]. Moreover, those who say they will definitely participate in a biobank are much more likely to say researchers don’t need to ask for permission (16%) or permission granted once only (28%). We did not assess subjects’ level of information on biobanks, but it cannot be excluded that people attending hospitals and who are involved in patients’ caregiving know more than the general public about research on biological materials. In addition, our data on IC preferences refer only to people who expressed willingness to participate in a biobank.

Public and ECs opinions also differ to some extent regarding the key information to be provided to the subjects [Table 3], with the public showing a tendency toward a greater interest in the aspects related to the relationship between researchers and donors, i.e., the authorisation to be re-contacted and to contact family members as well as the communication of research results. Subjects’ interest in the communication of research results is reiterated by almost all subjects’ desire to receive both individual results and research results of general value, while only around fifty per cent of ECs require results communication [Table 4]. This subjects’ expectation is confirmed in other surveys [19], returning research results is regarded as an influential factor in subjects’ decision to become biobank donors [20], and researchers endorse the obligation of communicating with research participants [21]. Anyway, there are a wide-ranging views and practices regarding the return of individual research results to participants [22]. Our participants’ request is in line with the ethical duty to return genetic research results of significance for the individual subject stated in international regulations [23], and with the subjects’ right to ask for their results of any type and to obtain them if they wish [24]. While ECs position may reflect the worry that the communication of research results that may be not informative enough for the individual subject and for which interpretation is difficult may generate misunderstanding and confusion for the subjects.

Family members are not really interested in information regarding sample property and IPR; in fact, around 70% of the subjects believe that the sponsor should be the owner of samples and IPR. On the contrary, those interviewed are greatly interested in having information about the sponsor’s prohibition on commercialising the samples and related data, which in addition they think should be given for free by the donors. Moreover, the large majority of the public regards the access of samples by not-for-profit organisations as acceptable, but only a minority agrees with access by for-profit organisations; on the contrary, ECs did not express a difference in opinion between the possibility of access from for-profit and not-for-profit organisations, both regarded as acceptable by around 70% of ECs. All these data converge in supporting a subjects’ disinterested view on their possible personal economic profit, that they ask be repaid with a similar approach by research promoters. The fact that people do not expect economic gain from participation in biobanks does not mean that they do not have an expectation of getting something in return [25]. From our sample’s responses, the desired reward seems to be primarily a good relationship with researchers who are asked to look at donors as active speakers who contribute to increasing present knowledge and the possibility of cure in the future and who are entitled to be informed of the research results and to be protected against discrimination.

### Limitations

The questionnaire on the attitudes towards genetic research was developed and used for the first time in this study, thus it was not validated in a previous and independent sample.

We did not formally check the participants’ level of knowledge and information on biobank. Therefore we could not evaluate if this influenced family members’ willingness to participate in a biobank constitution and their opinion on the ethical aspects of biobank management.

Participants were family members of patients attending a geriatric or neurological visit. At this stage of the project, patients were not involved in the survey. To investigate possible differences between the attitudes of patients and those of family members would be interesting, to evaluate how being affected by a disorders can shape willingness and opinions. Alternatively, a comparison among the attitudes of family members of patients affected from different kind of disorders could help to understand if different pathologies (affecting the brain or the body, with or without a possible treatment, for which we have or not knowledge of the causes) might influence people’s responses.

Finally, we acknowledge that our sample cannot be regarded as representative of the general public because it consists of the family members of patients suffering from a specific disturbance, and they were interviewed in an outpatient medical department. Both of these circumstances could influence their answers.

## Conclusion

We presented a survey conducted among the family members of patients who attended our institute for cognitive impairment or dementia to collect information on their views regarding biobank participation and management. We also collected elements to understand subjects’ attitudes towards medical research and genetic research and to delineate the features of the subjects available to take part in a biobank. The subjects’ and Italian ECs’ opinions on the ethical aspects of biobank management were then compared.

The so-called general public is made up of different publics invited to have a voice. In this sense, even though our sample is not representative of the general public, our subjects are one public and because they have a proximity with a disease that can be studied using genetic research and biobanks, they represent a public that is particularly qualified for expressing an opinion on the matter.

In the light of our findings, we believe that in developing biobank regulations, both ECs and policy makers should take into account not only expert opinions but also the views of lay people, on whom the possibility of the success of biobanks ultimately rests.

## Authors’ information

CP is the head of the Bioethics Unit at IRCCS Fatebenefratelli, a scientific institute for research and care in Alzheimer’s disease and mental disorders. PP is the head of the Medical Statistics and Information Technology Service at AFaR – Associazione Fatebenefratelli per la Ricerca. ET is a researcher at IRCCS Fatebenefratelli – Bioethics Unit. MP is the director of the Ethox Centre at Oxford University and an ethics advisor to UK Biobank.

## Electronic supplementary material

Additional file 1:**The questionnaires used in the survey are in Italian.** The questionnaire on the attitudes towards genetic research is provided as an additional file (Italian version and English translation). (ZIP 89 KB)
