# Supplementary material for: Public’s attitudes on participation in a biobank for research: an Italian survey
Source: BMC Med Ethics. 2014 Nov 26;15:81. doi: 10.1186/1472-6939-15-81 (PMC4258254; doi:10.1186/1472-6939-15-81)
Supplement: Supplementary file 1 — Additional file 1:The questionnaires used in the survey are in Italian. The questionnaire on the attitudes towards genetic research is provided as an additional file (Italian version and English translation). (ZIP 89 KB) [file 12910_2014_316_MOESM1_ESM.zip › 7671778541342889_add1.doc]

**Questionario 2: Ricerca Genetica**

1. Quale è la Sua attitudine verso la ricerca genetica?

 fortemente a sfavore  a sfavore  indifferente  a favore  fortemente a favore

2. Sarebbe disposto a partecipare a un progetto di ricerca genetica?

 Sì	 No

2.1 In caso di risposta affermativa, per quale motivo? (*è possibile indicare al max 2 risposte*):

a  accrescere la conoscenza/beneficio per le generazioni future

b  beneficio personale

c  beneficio per un familiare

d  “senso del dovere”

e  perché me lo richiede il medico che cura la mia malattia/quella del mio familiare

f  non c’è un motivo specifico

g  altro (specificare)

2.2 In caso di risposta negativa, per quale motivo? (*è possibile indicare max 2 risposte*):

a  timore nei confronti della ricerca genetica

b  timore di scoprire possibili predisposizioni genetiche a certe malattie

c  timore che non venga garantita la riservatezza

d  timore che le informazioni genetiche siano utilizzate per finalità discriminatorie

e  timore di un utilizzo del materiale genetico contrario ai miei principi

f  timore di utilizzo a fini commerciali del materiale genetico

g  paura degli aghi/punture etc.

h  non c’è un motivo specifico

i  altro (specificare)

3. Quale è la Sua attitudine verso la ricerca genetica riguardante i disturbi psichiatrici?

 fortemente a sfavore  a sfavore  indifferente  a favore  fortemente a favore

4. Sarebbe disposto a partecipare ad un progetto di ricerca genetica in ambito di disturbi psichiatrici?

 Sì	 No

4.1 In caso di risposta negativa, perché? (*è possibile indicare al max 2 risposte*):

a  timore di scoprire possibili predisposizioni genetiche a certe malattie

b  timore che non venga garantita la riservatezza

c  timore che le informazioni genetiche siano utilizzate per finalità discriminatorie

d  non c’è un motivo specifico

e  altro (specificare)

5. *da somministrare solo a famigliari che accompagnano pazienti per una visita psichiatrica*

Sarebbe d’accordo se il suo famigliare (paziente) partecipasse ad un progetto di ricerca genetica in ambito di disturbi psichiatrici?

 Sì	 No

5.1 In caso di risposta negativa, perché? (*è possibile indicare al max 2 risposte*):

a  timore di scoprire possibili predisposizioni genetiche a certe malattie

b  timore che non venga garantita la riservatezza

c  timore che le informazioni genetiche siano utilizzate per finalità discriminatorie

d  non c’è un motivo specifico

e  altro (specificare)

6. Quale è la Sua attitudine verso la ricerca genetica riguardante le demenze?

 fortemente a sfavore  a sfavore  indifferente  a favore  fortemente a favore

7. Sarebbe disposto a partecipare ad un progetto di ricerca genetica in ambito di demenze?

 Sì	 No

7.1 In caso di risposta negativa, perché? (*è possibile indicare al max 2 risposte*):

a  timore di scoprire possibili predisposizioni genetiche a certe malattie

b  timore che non venga garantita la riservatezza

c  timore che le informazioni genetiche siano utilizzate per finalità discriminatorie

d  non c’è un motivo specifico

e  altro (specificare)

8. *da somministrare solo a famigliari che accompagnano pazienti per una visita neurologica*

Sarebbe d’accordo se il suo famigliare (paziente) partecipasse ad un progetto di ricerca genetica in ambito di demenze?

 Sì	 No

8.1 In caso di risposta negativa, perché? (*è possibile indicare al max 2 risposte*):

a  timore di scoprire possibili predisposizioni genetiche a certe malattie

b  timore che non venga garantita la riservatezza

c  timore che le informazioni genetiche siano utilizzate per finalità discriminatorie

d  non c’è un motivo specifico

e  altro (specificare)
