# Supplementary material for: Public’s attitudes on participation in a biobank for research: an Italian survey
Source: BMC Med Ethics. 2014 Nov 26;15:81. doi: 10.1186/1472-6939-15-81 (PMC4258254; doi:10.1186/1472-6939-15-81)
Supplement: Supplementary file 1 — Additional file 1:The questionnaires used in the survey are in Italian. The questionnaire on the attitudes towards genetic research is provided as an additional file (Italian version and English translation). (ZIP 89 KB) [file 12910_2014_316_MOESM1_ESM.zip › 7671778541342889_add2.doc]

**Questionnaire 2: Genetic Research**

1. What is your attitude towards genetic research?

 strongly disagree  disagree  neutral  agree  strongly agree

2. Would you be willing to participate in a genetic research project?

 Yes	 No

2.1 If yes, why? *(it is possible to choose maximum 2 answers)*:

a  to increase knowledge / benefit for future generations

b  personal benefit

c  benefit for a family member

d  sense of duty

e  because my physician / my family member’s physician asks me to participate

f  no specific reason

g  other (please, specify)

2.2 If not, why? *(it is possible to choose maximum 2 answers*)

a  worry about genetics research

b  worry to discover possible genetic predispositions to some diseases

c  worry about a break of confidentiality

d  worry that genetic information may be used for discriminatory purposes

e  worry that genetic materials may be used against my principles

f  worry that genetic materials may be used for commercial purposes

g  I am afraid of needles/injections

h  no specific reason

i  other (please, specify)

3. What is your attitude towards genetic research on psychiatric disorders?

 strongly disagree  disagree  neutral  agree  strongly agree

4. Would you be willing to participate in a genetic research on psychiatric disorders?

 Yes	 No

4.1 If not, why? *(it is possible to choose maximum 2 answers):*

a  worry to discover possible genetic predispositions to some diseases

b  worry about a break of confidentiality

c  worry that genetic information may be used for discriminatory purposes

d  no specific reason

e  other (please, specify)

5. *To administer only to family members of patients attending a psychiatric visit*

Would you agree with the patient’s participation in a genetic research project on psychiatric disorders?

 Yes	 No

5.1 If not, why? *(it is possible to choose maximum 2 answers):*

a  worry to discover possible genetic predispositions to some diseases

b  worry about a break of confidentiality

c  worry that genetic information may be used for discriminatory purposes

d  no specific reason

e  other (please, specify)

6. What is your attitude towards genetic research on dementia?

 strongly disagree  disagree  neutral  agree  strongly agree

7. Would you be willing to participate in a genetic research project on dementia?

 Yes	 No

7.1 If not, why? *(it is possible to choose maximum 2 answers):*

a  worry to discover possible genetic predispositions to some diseases

b  worry about a break of confidentiality

c  worry that genetic information may be used for discriminatory purposes

d  no specific reason

e  other (please, specify)

8. *To administer only to family members of patients attending a neurological visit*

Would you agree with the patient’s participation in a genetic research project on dementia?

 Yes	 No

8.1 If not, why? *(it is possible to choose maximum 2 answers):*

a  worry to discover possible genetic predispositions to some diseases

b  worry about a break of confidentiality

c  worry that genetic information may be used for discriminatory purposes

d  no specific reason

e  other (please, specify)
